# Fine microstructure formation in steel under ultrafast heating

**DOI:** 10.1038/s41598-019-47668-6

**Published:** 2019-08-02

**Authors:** Mitsuharu Yonemura, Hitomi Nishibata, Tomohiro Nishiura, Natsumi Ooura, Yuki Yoshimoto, Kazuki Fujiwara, Kaori Kawano, Tomoyuki Terai, Yuichi Inubushi, Ichiro Inoue, Kensuke Tono, Makina Yabashi

**Affiliations:** 1 Advanced Technology Research Laboratories, Nippon Steel Corporation, 1-8 Fuso-cho, Amagasaki, Hyogo 660-0891 Japan; 2 Department of Materials Science and Engineering, Graduate School of Engineering, Osaka University, 2-1 Yamadaoka, Suita, Osaka 565-0871 Japan; 3 Japan Synchrotron Radiation Research Institute, 1-1-1 Kouto Sayo-cho Sayo-gun, Hyogo, 679-5198 Japan; 4 RIKEN SPring-8 Center, 1-1-1 Kouto Sayo-cho Sayo-gun, Hyogo, 679-5148 Japan

**Keywords:** Metals and alloys, Characterization and analytical techniques

## Abstract

In this study, phase transformation kinetics was directly evaluated using a femtosecond X-ray diffraction technique for operand measurements of the dislocation densities and carbon concentrations in Fe-0.1mass%C martensitic steel. To identify the reverse transformation mechanism from α′ to γ, we used an X-ray free-electron laser and ultrafast heating. A maximum heating rate of 10^4^ °C/s, which is sufficient to avoid diffusive reversion, was achieved, and the reverse transformation during ultrafast heating was successfully observed. Our results demonstrated that a fine microstructure formed because of a phase transformation in which the dislocation density and carbon concentrations remained high owing to ultrafast heating. Fe–C martensitic steels were also found to undergo a massive reverse transformation during ultrafast heating. The formation of a fine microstructure by a simple manufacturing process, without rare elements such as Ti, Nb, or Mo, can be expected. This study will help further the development of functional steels.

## Introduction

Annual global steel production has been increasing owing to a growing demand for high-strength steels that enable automobiles to be designed with thinner and therefore lighter parts that help to reduce fuel consumption and reduce the global carbon footprint. Advanced high-strength steels have been conventionally developed by optimizing alloying elements and heat treatments. Among the various strengthening mechanisms used for advanced steels, grain refinement is one of the few methods that can improve both strength and toughness simultaneously^1^. Therefore, ultrafine grained steels have great potential for replacing some conventional advanced steels. The grain refinement of steel on the nanometer scale has been investigated through heavy deformation, such as high pressure torsion^2^ and accumulated roll bonding^3^, at low temperature. However, the application of heavy deformation at low temperature naturally increases the deformation load while tending to decrease the productivity of the grain-refinement processes used. On the other hand, the metallurgy of steel produced using hot rolling and subsequent accelerated cooling in hot-strip and plate mills has been studied as thermo-mechanical controlled processes (TMCPs) for several decades^4^, and the formation of nanometer-scale grains during production through control of the cooling rate has been extensively studied^5–7^. This nanograin refinement is due to the rapid-cooling promoted enhanced nucleation of ferrite in austenite with accumulated and frozen-in strain. However, these techniques cannot be applied to products that require reheating, such as cold-rolled steel and plated steel sheet, since their ultrafine structures will be coarsened during the reheating process. A recent publication suggested that applying ultrafast heating to cold-rolled low-alloy steels results in a substantial grain refinement^8^. Several studies have focused on improving both the strength and ductility of low-carbon steel through grain refinement by rapid heating^8–13^. Petrov^14,15^ reported that the average grain size drops from 5 μm to 1 μm as the heating rate is increased from 10^2^ °C s^−1^ to 10^3^ °C s^−1^ in cold-rolled HSLA steels and DP steels, resulting in an increase in tensile strength. Ferrite-austenite transformations occur in the recrystallized matrix during conventional heating at a rate lower than 10 °C s^−1^. On the other hand, the microstructures of samples that were heated at rates as high as 3000 °C s^−1^ and subsequently quenched suggest that both recovery and recrystallization were completely suppressed and that the transformation from ferrite to austenite began before the onset of recrystallization. Therefore refinement is considered to be influenced by the kinetics of recovery and the driving force for ferrite-to-austenite transformation. However, it is impossible to observe atomic diffusion and recovery during ultrafast microstructural changes in ultrafast quenched carbon steels, which has hindered the physical and kinetic understanding of these processes.

Furthermore, whether the transformation mechanism from ferrite to austenite is diffusive or displacive is controversial. Researchers have systematically observed microstructural changes in quenched materials heated at 1000 °C s^−1^ ^16–20^ and have modeled the displacive phase transformation between ferrite and austenite^21^. Despite these studies and many other efforts, a complete understanding of the microstructural changes under ultrafast heating has remained elusive because of the lack of operand measurements capable of providing information about martensitic transformations. To date, microstructural changes under ultrafast heating (i.e., heating faster than 10^4^ °C s^−1^) have not been directly observed.

We also discuss the effects of ultrafast heating on microstructural formation within the context of dislocation migration and carbon diffusion.

## Methods

### Experimental setup

Single-shot X-ray diffractometry (XRD) is a highly effective method for observing rapid and irreversible microstructural changes. Synchrotron-radiation facilities produce X-rays that are so bright that diffraction patterns have been recorded with exposure times as short as ~10 ms^22,23^. However, such exposure times are far longer than the target temporal resolution for the direct observation of rapid microstructural changes in iron and steel (<1 μs). Therefore, significantly more intense X-ray beams are required, with flux densities at least four-orders-of-magnitude greater. At present, these types of intense X-ray beams are produced by X-ray free-electron lasers (XFELs)^24^.

In this study, time-resolved XRD was performed using an XFEL at the SPring-8 Angstrom Compact free-electron Laser (SACLA, Hyogo, Japan)^25,26^ in order to clarify changes in the dislocation densities and carbon concentrations during martensitic transformations at ultrafast heating rates up to and exceeding 10^4^ °C s^−1^, which is much higher than that claimed for previous operand techniques^22,23^ and makes hitherto unexplored regimes of non-equilibrium states accessible.

**Figure 1:**
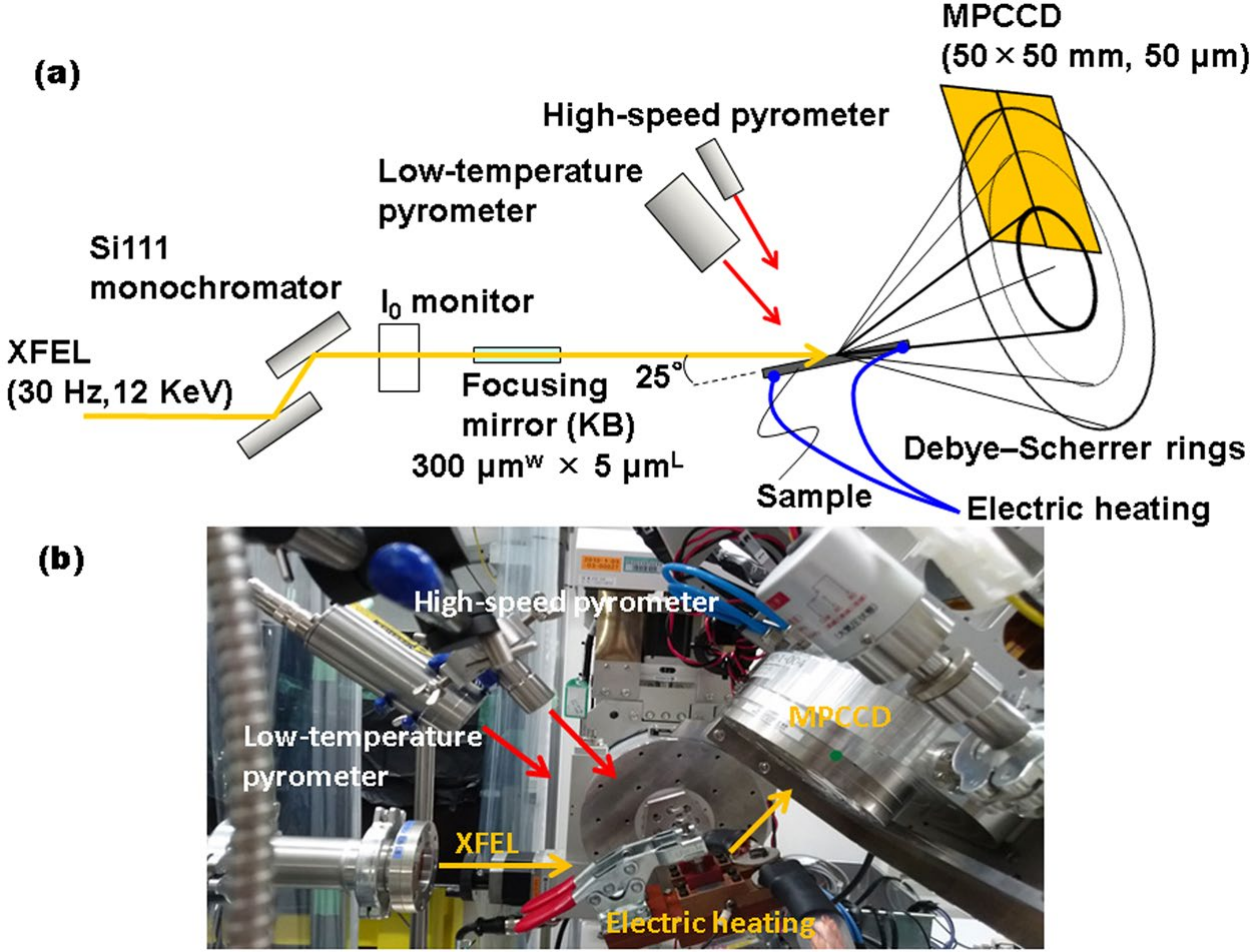
Experimental setup for operand measurements during ultrafast heating. (**a**) Schematic illustration and (**b**) photographic image around the sample.

To clarify the phase transformations during ultrafast heating, femtosecond XRD was performed using an XFEL. Figure 1 illustrates the experimental setup. The incident beam was monochromatized with a Si(111) monochromator to achieve an energy width of approximately 1 eV (full-width at half-maximum: FWHM). The beam was focused with an elliptical mirror to 300 μm (horizontal direction) × 7 μm (vertical) at the sample. The divergence angle in the vertical direction was 0.1 mrad and the glancing angle was 25°. A photon energy of 12 keV (i.e., a wavelength of 0.103333 nm) was selected to acquire a sufficient number of diffraction peaks for the α and γ phases, without overlap. For electrical heating, temperatures were measured using a high-speed pyrometer, whose emissivity was corrected to 0.9 by simultaneous measurements with a type-R thermal couple and a two-color pyrometer at a lower heating rate, with a 10 μs time resolution and 400 μm spot size. The emissivity is influenced by material and surface contaminants, such as oxidation products. The temperature measurement is completed without oxidation at high temperature through ultrafast heating; in fact, no oxide-related diffraction was observed. Furthermore, the emissivity of iron has a low temperature dependence, since the pyrometer operates at a wavelength of 2.0–2.5 μm^27^. Therefore, the emissivity is constant at the temperatures in this study.

X-ray detection was performed with a multiport charge-coupled device (MPCCD)^28^, and the heating system was controlled via a trigger signal from SACLA with the electrical heating condition. Both edges of the sample were held by a copper electrode for electrical heating. The heated area of the sample was 15 mm × 5 mm × 0.5 mm. To suppress the effects of crystal orientation, diffraction patterns were acquired in a wide reciprocal lattice space by using an MPCCD detector with two sensor modules at a camera length of 150 mm and angles of 35°, 55°, and 77° from the horizontal. Heating and temperature measurements began by the edge-triggering of the transistor-transistor-logic (TTL) level that is delayed from the Open gate signal of the XFEL at setup time. On the other hand, the diffraction measurements are synchronized with the Open gate signal. The TTL is controlled on the nanosecond timescale, which is faster than the heating rate. The XFEL frequency was 30 Hz, which corresponded to a temperature step of 333 °C at a heating rate of 10^4^ °C s^−1^. We achieved a temperature resolution of 100 °C or less by delaying the timing of the TTL via the Open gate signal. The sample was replaced with a new one after every heating cycle. Eleven measurements were taken to ensure statistical significance of the data. For each sample, 100 dark images were recorded for background correction. Additionally, to calibrate the diffraction angles, 100 diffraction images were collected before heating. Further, XRD measurements at a heating rate of 2 °C s^−1^ were performed in a vacuum using a sample heating stage (DHS1100, Anton Paar) and X-ray diffractometer (SmartLab, Rigaku).

### X-ray line profile analysis

XRD line profiles were obtained by integrating diffraction images in the circumferential direction. We applied an X-ray line profile analysis (XLPA) originally developed by Williamson and Hall^29^ and Warren and Averbach^30^ in the 1950s. In the theory proposed by Ungár *et al*.^31,32^ in the 1980s, line profiles are analyzed by considering: 1) the effects of anisotropic lattice strains on crystallographic orientations, and 2) the strength of the lattice strains around dislocations. These characteristics are used to deduce the optimal relationship between dislocation density and the X-ray line profiles. Here, large lattice strains occur in specific crystallographic orientations because Burgers vector depends on the crystal system^33^. From the modified Williamson–Hall and modified Warren–Averbach^31^ procedures using a mean contrast factor based on elastic anisotropy, the XLPA allows us to evaluate the properties of the substructure, such as the dislocation density, fraction of the edge/screw dislocation, and dislocation arrangement.

The FWHM of the normalized peaks can be evaluated by the modified Williamson–Hall plot as Equation (1):

1 $${\rm{\Delta }}K=0.9/D+{(\pi {M}^{2}{b}^{2}/2)}^{1/2}\cdot {\rho }^{1/2}K{C}^{1/2}+O({K}^{2}C)$$

where $K=2sin\theta /\lambda$ and ${\rm{\Delta }}K=2cos\theta ({\rm{\Delta }}\theta )/\lambda$ is the magnitude of the FWHM*. θ* is the diffraction angle and *λ* is the X-ray wavelength. *D, ρ*, and *b* are the average particle size, average dislocation density, and Burgers vector, respectively. *M* is a constant that varies depending on the effective outer cut-off radius of the dislocations. *O* indicates the non-interpreted higher-order terms.

Now, the dislocation density can be obtained by the modified Warren–Averbach method as equation (2):

2 $$lnA(L)=ln{A}^{s}(L)-\rho \cdot \frac{\pi {b}^{2}}{2}\cdot {L}^{2}\cdot ln(\frac{{R}_{e}}{L})\cdot ({K}^{2}C)+Q({K}^{4}{C}^{2})$$

where *A*(*L*) is the real part of the Fourier coefficients, *A*^*s*^ is the size Fourier coefficient as defined by Warren, *R*_e_ is the effective cut-off radius of the dislocations according to the output parameter for each analysis, and *Q* represents the second-order terms of *K*^2^*C*. *L* is the Fourier length. Then, *Q* indicates the non-interpreted higher-order terms.

Here, the contrast factor *C* depends on the average contrast factor *C*_*h*00_, the parameter of the lattice index (*h*, *k*, *l*) *H*, and *q* depending on the elastic constants of the crystal and the characteristics of the dislocations, i.e., the screw/edge fraction, in the crystals as Equation (3):

3 $$C={C}_{h00}(1-q{H}^{2})$$

where $${H}^{2}=({h}^{2}{k}^{2}+{h}^{2}{l}^{2}+{k}^{2}{l}^{2})/{({h}^{2}+{k}^{2}+{l}^{2})}^{2}.$$

Using Equations (1), (2) and (3), *ρ*, *q*, and *D* were analyzed.

If the experimentally determined *q*_*exp*_ is close to the theoretical value calculated for an edge or screw dislocation (*q*_*edge*_ or *q*_*screw*_, respectively), the character of the dislocation structure is edge- or screw-like; however, the character of the dislocation is mixed when *q*_*exp*_ lies between *q*_*edge*_ and *q*_*screw*_. The screw-character degree of the dislocation can be described by the quantity^34^:

4 $$({q}_{exp}-{q}_{edge})/({q}_{screw}-{q}_{edge})$$

which is zero or unity for a pure edge or screw dislocation, respectively. The constants and parameters for these calculations are listed in Table 1.

**Table 1:** Parameters and constants for calculations at room temperature.

Phase	*C*_*h*00_ (screw/edge)^34^	*q* (screw/edge)^34^	Burgers vector (nm)	Shear modulus (N/m^2^)
martensite	0.301/0.255	2.67/1.28	0.248	8.0 × 10^10^
austenite	0.265/0.265	2.21/1.38	0.249	7.5 × 10^10^

To determine the diffraction angle and FWHM, the asymmetric X-ray line profiles were fitted with a split pseudo-Voigt function^35^. Furthermore, geometry errors in the XRD measurements were corrected by comparing diffraction data for the sample (before heating) to reference data from the NIST standard (660c).

Further, we calculated the elastic strain energy (*E*_*dis*_) from the measured dislocation density (*ρ*) and the effective cut-off radius of dislocation (*R*_e_) by following a well-known equation^36^:

5 $${E}_{dis}=\frac{\mu {b}^{2}}{4\pi }ln\frac{{R}_{e}}{r}\cdot \rho $$

where μ is the shear modulus, and *r* is the radius of the dislocation core. Generally, r is 5*b*.

Table 1 lists the constants or other parameters assumed and used during the calculations of dislocation density and elastic strain energy, at room temperature as an example.

## Results and Discussion

### Operand measurement during ultrafast heating

**Figure 2:**
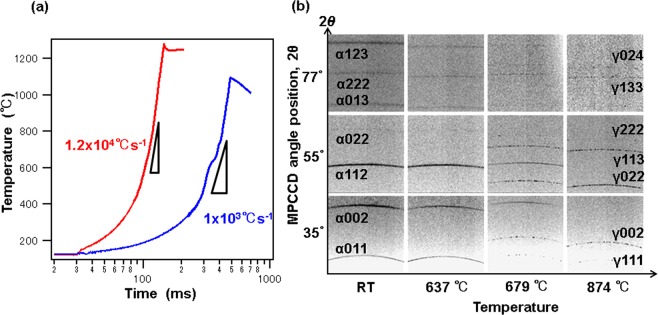
Heating curve and some diffraction patterns. (**a**) Heating curve acquired with a monochromatic pyrometer. (**b**) Diffraction patterns at a heating rate of 1.2 × 10^4^ °C s^−1^.

Figure 2(a) shows heating curves for the sample under two different heating conditions. The sample was prepared with a composition of Fe-0.1mass%C-2mass%Mn having a martensitic microstructure and was cold-rolled to reduce its thickness by 50%. The average heating rates between 500 and 1000 °C were 1 × 10^3^ and 1.2 × 10^4^ °C s^−1^.

Figure 2(b) shows the XRD patterns recorded for a heating rate of 1.2 × 10^4^ °C s^−1^. Each pattern was obtained from a single-pulse XFEL exposure with a duration of about 10 fs. The detector was positioned at 35°, 55°, or 77°. Debye–Scherrer rings of the α phase clearly appear at room temperature, while those of the γ phase emerge at 679 °C (Ac_1_). The XRD patterns clearly exhibit a reverse transformation from the α to the γ phase at 679 °C (Ac_1_). Furthermore, we observe a sharpening and shifting of the Debye–Scherrer rings with the increase in temperature. The ring patterns of the γ phase, which appear spotty, indicate uniform nucleation during the phase transformation. Full austenite forms at approximately 874 °C (Ac_3_).

### Change in dislocation density and characteristics during ultrafast heating

**Figure 3:**
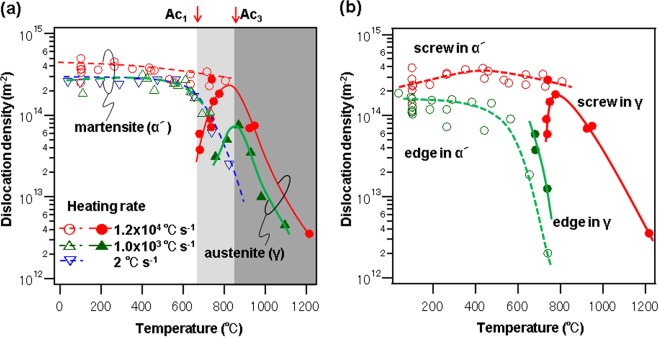
Dislocation density and characteristics as functions of temperature. (**a**) Temperature dependence of the dislocation density at different heating rates. (**b**) Temperature dependence of the screw and edge dislocation densities at 1.2 × 10^4^ °C s^−1^.

Figure 3(a) shows the changes in dislocation density values for different heating rates. The dashed and solid lines represent the martensite (α′) and austenite (γ) phases, respectively. The red, green, and blue lines represent the dislocation densities at heating rates of 1.2 × 10^4^, 1 × 10^3^, and 2 °C s^−1^, respectively. A two-phase zone containing both the α′ and γ phases exists between 680 °C (Ac_1_) and 820 °C (Ac_3_) at a heating rate of 1.2 × 10^4^ °C s^−1^. The dislocation density of the α′ phase decreases from 3 × 10^14^ m^−2^ at about 600 °C to 3 × 10^13^ m^−2^ at about 800 °C at a heating rate of 2 °C s^−1^, indicating recovery of the microstructure. Similarly, the dislocation density of the α′ phase at a heating rate of 10^3^ °C s^−1^ also decreases. In contrast, the dislocation density of the α′ phase at a heating rate of 1.2 × 10^4^ °C s^−1^ is constant even in the high-temperature region, where the α′ phase transforms into the γ phase. In the γ phase above Ac_1_, the dislocation density is found to be relatively low at the onset of the reverse transformation at Ac_1_. The dislocation density in the γ phase increases as the γ phase grows and reaches its maximum value around Ac_3_, where the full austenite is formed. Beyond Ac_3_, the dislocation density significantly decreases to 10^12^ m^−2^. This value corresponds to a large elastic strain energy of 1.7 MJ m^−3^. At high temperatures, Yoshie *et al*. have reported similar γ-phase dislocation densities in 0.1mass%C-1.4mass%Mn-bal.Fe steel^37^. Furthermore, the α′ phase and γ phase coexist at least between 680 °C and 820 °C. The Ac_3_ temperature at a heating rate of 1.2 × 10^4^ °C s^−1^ is almost identical to that at a heating rate of 1.0 × 10^3^ °C s^−1^. The maximum dislocation density of the γ phase is lower for the lower heating rate. This result indicates that slower heating allows the coalescence and annihilation of the dislocations to proceed at the high temperature, where the crystal growth rate is relatively low. The γ phase was not clearly observed in laboratory measurements at the low heating rate of 2 °C s^−1^, because the sample temperature was limited below about 800 °C.

Figure 3(b) illustrates the temperature dependence of the dislocation densities for the screw and edge components at a heating rate of 1.2 × 10^4^ °C s^−1^. The dashed and solid lines represent the martensite (α′) and austenite (γ) phases, respectively. The red and green lines represent the screw and edge dislocation components, respectively. Although the total dislocation density is approximately 4 × 10^14^ m^−2^ below 500 °C, as shown in Fig. 2(a), the edge dislocation component slightly decreases from 2 × 10^14^ to 1 × 10^14^ m^−2^ as the screw dislocation component increases from 2 × 10^14^ to 4 × 10^14^ m^−2^. This behavior originates from the deformation of the dislocation loop, which occurs when an edge dislocation with high mobility migrates rapidly. The edge dislocation component decreases drastically to 2 × 10^12^ m^−2^ in the region above 600 °C. In contrast, the screw dislocation component decreases only slightly. Therefore, the screw dislocation is the main component in the α′ phase at high temperatures (i.e., temperatures above Ac_1_). In contrast, edge dislocations are dominant in the γ phase around Ac_1_. Subsequently, as the temperature rises, the characteristic of the dislocation immediately changes from edge-type to screw-type, implying that edge dislocations are unstable at high temperatures. The screw dislocation component of the γ phase decreases rapidly in full austenite at Ac_3_. This phenomenon corresponds to the ledge growth observed with transmission electron microscopy (TEM) by Kinsman^38^ and Purdy^39^ during the initial precipitation stages. Instead of an unstable edge dislocation at high temperatures, numerous screw dislocations are introduced to the matrix to promote three-dimensional screw growth. The γ-phase dislocation multiplication in the two-phase zone thus results in the crystal growth of the γ phase.

**Figure 4:**
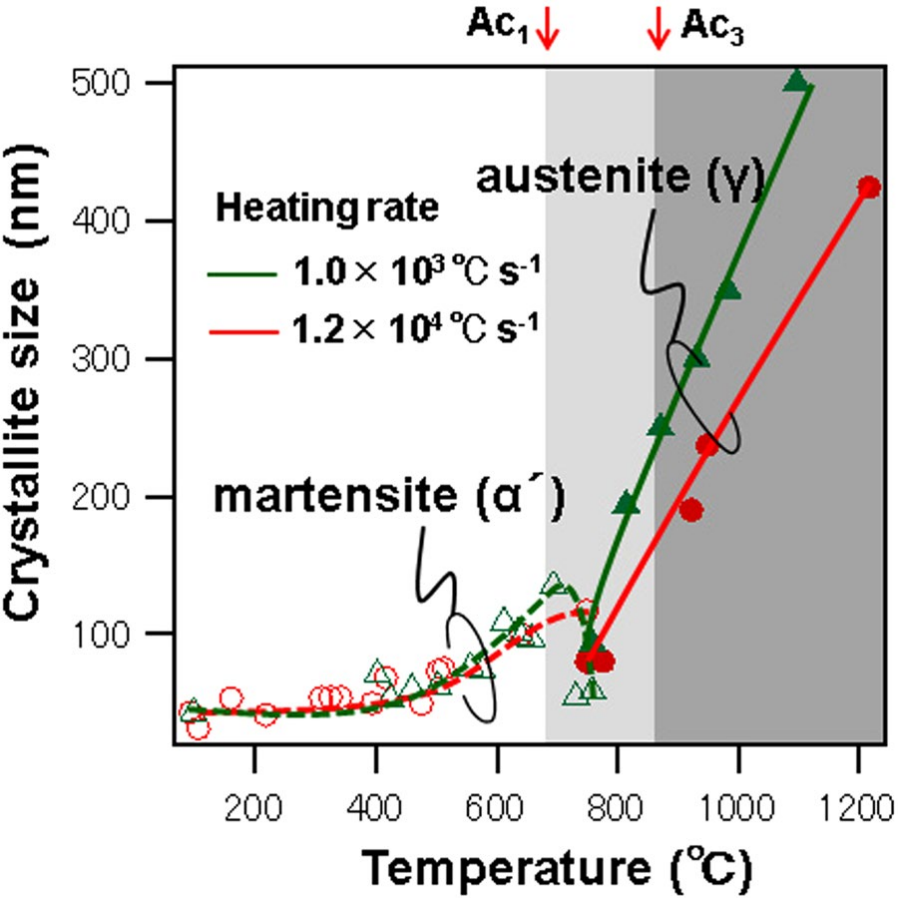
Temperature dependence of crystallite size on heating rate. The dashed and solid lines represent the martensite (α′) and austenite (γ) phases, respectively. The red and green lines represent heating rates of 1.2 × 10^4^ and 1 × 10^3^ °C s^−1^, respectively.

Figure 4 shows the temperature dependence of the crystallite sizes at different heating rates. The crystallite size of the α′ phase increases as the temperature rises to Ac_1_ at both heating rates. The α′ crystallite size decreases in the two-phase zone at a heating rate of 1.0 × 10^3^ °C s^−1^, presumably because of the separation of the α′ phase by the nucleation of the γ phase. In contrast, this type of decreasing trend was not observed at the heating rate of 1.2 × 10^4^ °C s^−1^. This phenomenon is likely attributed to ultrafast growth from high-density nucleation, which results in the formation of fine crystal grains in the γ phase. In the case of the γ phase, the growth curves at the two heating rates are clearly different. Crystallite growth is suppressed at the higher heating rate, which results in the formation of finer crystal grains.

### Change in carbon concentration during ultrafast heating

The carbon concentration in the α′ phase during heating was also estimated from the Fe–C lattice constants, which were determined by analyzing X-ray line profiles. Generally, the carbon concentrations change significantly, even in response to only slight variations in the Fe–C lattice constant^40^. Therefore, to estimate the carbon concentrations, the lattice constants should be determined precisely. Martensitic steel less than 0.2mass%C has a body-centered-cubic (BCC) crystal lattice. As we also experimentally confirmed that the fraction of c/a is equal to unity, the dislocation in the BCC structure is herein discussed.

**Figure 5:**
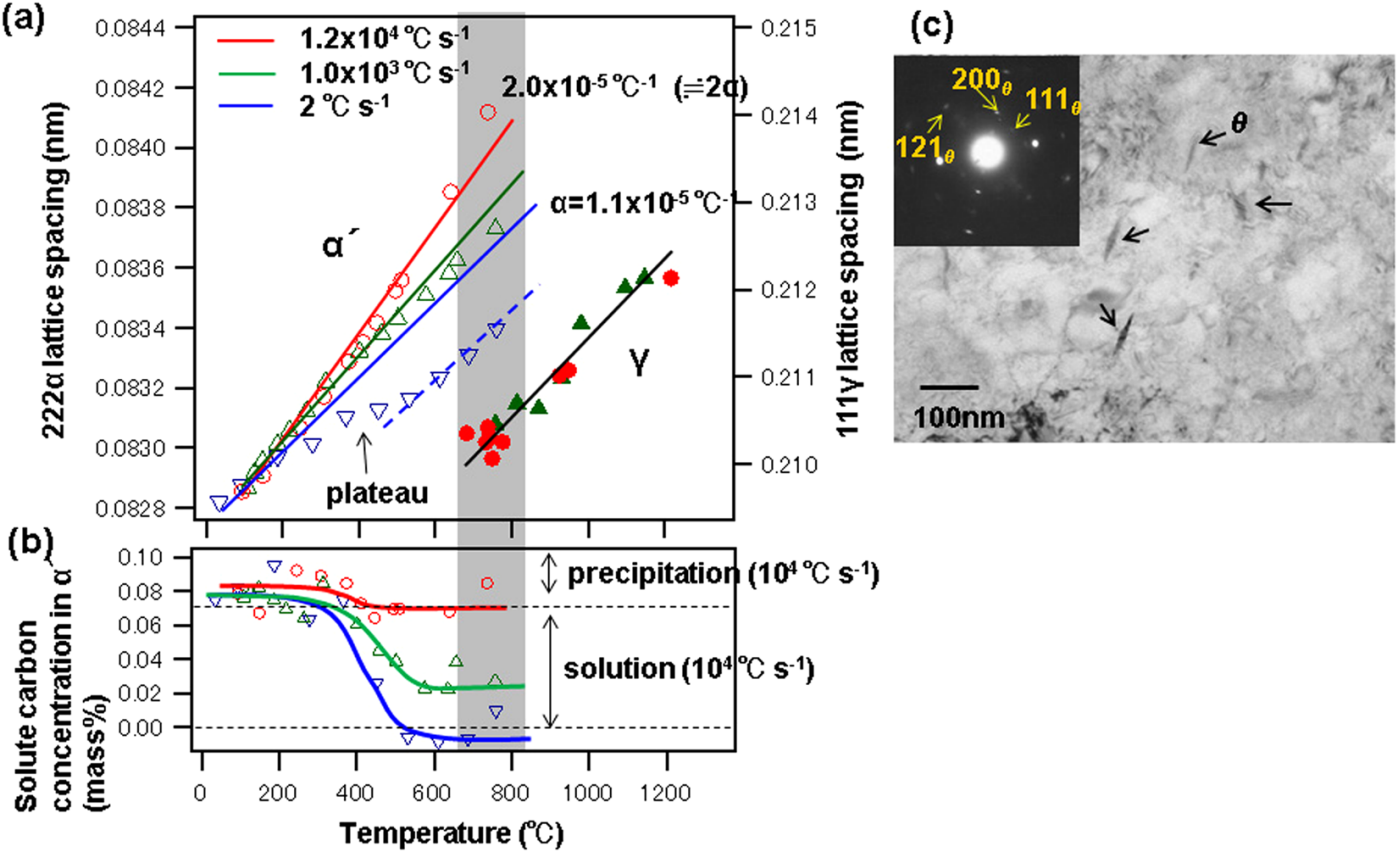
Temperature dependences of lattice spacing and carbon concentration for the α′ phase. (**a**) Displacement of the 222 α and 111 γ lattice spacings with temperature. (**b**) Carbon concentrations at different heating rates. (**c**) TEM images of the initial microstructure. Needle-like θ-Fe_3_C precipitates several tens of nanometers in length are observed.

Figure 5(a,b) illustrate the temperature dependence of the lattice spacing and carbon concentrations in the α′ phase for different heating rates. Red, green, and blue lines represent changes in the {222} lattice constant for the α′ phase (left axis) at heating rates of 1.2 × 10^4^, 1 × 10^3^, and 2 °C s^−1^, respectively. The black line represents the change in the lattice constant of {111} in the γ phase (right axis). Lattice spacings of {111} for the γ phase and {222} for the α′ phase (which were not affected by lattice strain multiplicity) were used to estimate the thermal expansion coefficient. The lattice spacing of the α′ phase increases linearly from room temperature as the temperature rises. For a heating rate of 2 °C s^−1^, the plateau at about 400 °C indicates a decrease in carbon concentration. This plateau does not clearly appear at higher heating rates, i.e., a slight decrease in carbon content. We determined the thermal expansion coefficient for each heating rate. The value at 2 °C s^−1^ (1.1 × 10^−5^ °C^−1^) agrees with the value for carbon steel^41^. The thermal expansion coefficient increases with the heating rate. At 1.2 × 10^4^ °C s^−1^, the value of the coefficient is approximately twice as large as it is at 2 °C s^−1^. It is well-known that dislocations in BCC crystals strongly depend on the strain rate, because BCC dislocations do not migrate effectively at low temperatures^42,43^. Therefore, lattice spacings strongly depend on the heating rate in the range from room temperature to Ac_1_. As the heating electrodes at both ends of the restricted sample expand horizontally, the sample expands vertically from the surface. Even under such conditions, the lattice spacing continues to increase linearly with temperature. This result implies an elastic rather than plastic sample response. The elastic zone can be wide because plastic deformation is difficult in cases where the transformation stress does not relax with dislocation migration. This was interpreted as a difference in the temperature dependence of the lattice spacing.

In contrast, the γ-phase lattice spacing increases monotonically and is not dependent on the heating rate. The samples deform elastically because of thermal stresses regardless of the phase transformation, but undergo little plastic deformation. It has been reported that introducing dislocations does not significantly affect plastic deformation^44^. Thermal stress is well-known to be a function of temperature but not heating rate. At different heating rates, the figure shows linear relationships between the sample lattice spacing and temperature. Even if both ends of the sample are restricted, the γ-phase nucleus effectively relieves thermal stress because the thermal expansion is strongly dependent on crystal orientation^45^. As the width direction (W) and length direction (L) of the thin sample are restricted, it is supposed that only the heat strain (εW, εL) is biaxially compressed in these directions. Thus, the strain of the thickness direction becomes 0.3 εW + 0.3 εL because Poisson’s ratio is 0.3 for mild steel^46^. Moreover, by isotropic thermal expansion, the coefficient of thermal expansion is 0.6 α. The expansion of the lattice plane becomes 1.6 α by adding thermal expansion α in the thickness direction. A coefficient of thermal expansion of 2 α in this study implies a Poisson ratio of nearly 0.5. Though Poisson’s ratio of mild steel is known to slightly increase with temperature^47^, ultrafast heating may introduce special behavior such as an increase in the Poisson’s ratio.

Multiple γ-phase nuclei continually emerge from the α phase, and the γ-phase fractions increase as the crystals grow with temperature. Consequently, the lattice spacing of the γ phase exhibits the same temperature dependence at different heating rates. Of course, the thermal expansion should depend on the heating rate in full austenite above Ac_3_. However, no clear difference is observed, as dislocations in the face-centered-cubic (FCC) lattice migrate sufficiently rapidly at high temperatures^48,49^. The dislocation migration must overcome potential barriers in the matrix. The frequency for overcoming the potential barriers corresponds to the dislocation migration rate, which depends on the crystallographic structure and temperature^50^. The migration rate in BCC crystals is exponentially dependent on temperature. Therefore, dislocation migration cannot follow thermal stress at low temperatures (i.e., temperatures below Ac_1_). In contrast, because the potential barrier in the FCC phase is lower, dislocations in the full austenite can easily migrate at high temperatures above Ac_3_ to relieve thermal stress.

We estimated the apparent lattice spacing of {222}α at room temperature by extrapolating the acquired expansion curves; {222}α does not influence the reflection multiplicity. That is, we can measure precisely the lattice constant even if the c/a ratio changes from unity. Carbon concentrations were calculated from the apparent lattice spacing by applying Vegard’s law of carbon content between 0.82769 nm as the lattice spacing of the Fe-2mass%Mn alloy and 0.82856 nm as that of the Fe-2mass%Mn-0.1mass%C alloy^51,52^. In this calculation, a cubic system was assumed because the c/a ratio was approximately constant in the tetragonal system. Figure 5(b) shows the solute carbon concentrations in the α′ phase at three heating rates. The red, green, and blue lines represent the solute carbon concentrations in the α′ phase at heating rates of 1.2 × 10^4^, 1 × 10^3^, and 2 °C s^−1^, respectively. The carbon concentrations were calculated to be about 0.08 mass% at low temperatures, slightly lower than the nominal value of 0.1 mass% C. As shown in the TEM images of the initial microstructure in Fig. 5(c), we observe needle-like θ-Fe_3_C precipitates several tens of nanometers in length. As some carbon in the matrix formed θ-Fe_3_C, whose carbon content is 6.69 mass%, it is considered that the carbon content decreases in the matrix. The carbon concentrations decrease in the region above 300 °C because of θ-Fe_3_C precipitation in the matrix, as shown in Fig. 5(c). The rate of decrease is higher at lower heating rates. The carbon concentrations at 2 °C s^−1^ drop to zero, indicating that carbon atoms are fully taken into the precipitation of the θ-Fe_3_C in the matrix. The reduction in carbon concentrations is suppressed by increasing the heating rate. This result suggests that ultrafast heating facilitates the formation of microstructures with high carbon concentrations. Therefore, the nucleation of the γ phase is promoted at the high heating rate of 1.2 × 10^4^ °C s^−1^, producing fine crystal grains. The slight decrease during the initial stage (below 400 °C) suggests that a small amount of carbon atoms may contribute to the precipitation and coarsening of θ-Fe_3_C in the initial microstructure by short-range diffusion. The carbon concentration in the γ phase is approximately constant and does not depend on the heating rates, because the lattice spacing shows a linear temperature dependence (Fig. 5(a)). At each heating rate, the solute carbon concentrations in the α′ phase are approximately constant in the two-phase zone. These results imply a massive transformation with the ledge growth of the γ grains^53^. That is, lattice rearrangement occurs because of short-range diffusion at the α/γ interface with the introduction of misfit edge dislocations. Reverse transformation has been proposed to be massive at heating rates above 1 × 10^3^ °C s^−1^ in pure iron^54,55^.

### Fine microstructure formation

**Figure 6:**
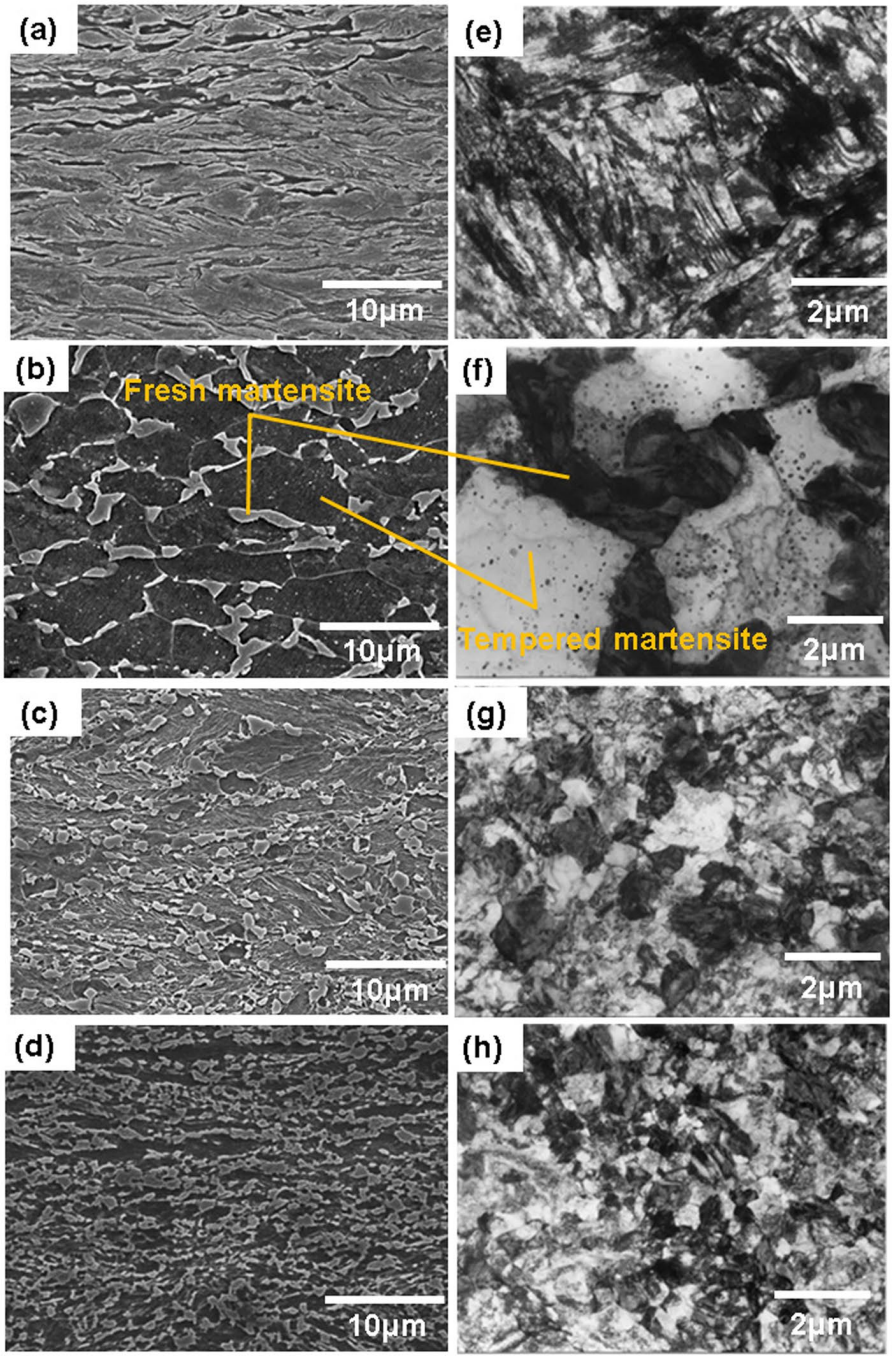
SEM images (left) and TEM images (right) of (**a**) and (**e**) the initial microstructure, and the microstructures obtained by quenching the samples with water at 800 °C at heating rates of (**b**) and (**f**) 2, (**c**) and (**g**) 10^3^, and (**d**) and (**h**) 10^4^ °C s^−1^.

The short-range diffusion transformation at the high heating rate causes the formation of fine crystal grains, in comparison to the diffusion transformation at the low heating rate, as shown in Fig. 6(b,f). As a typical example of shear transformation, the Fe-30mass%Ni produces a flat α/γ interface caused by the migration of the habit plane^56^. In contrast, at high heating rates, the α′/prior-γ-grain boundary shows an irregular patch, which is characteristic of massive grains, as shown in Fig. 6(d,h).

In the α′ matrix, dislocation recovery has been delayed to higher temperatures, and the carbon concentration in α′ increases with heating rate. This suggests that the ferrite matrix, which maintains a high density of dislocations and super-saturated C atoms, might transform to austenite in an allotropic manner with the short-range diffusion of Fe and C atoms. These characteristics are observed experimentally at heating rates of up to 10^3^ °C s^−1^ in the SCM435 alloy^16^. This type of non-diffusive behavior becomes more remarkable at higher heating rates of 10^4^ °C s^−1^, at which point the diffusion distance of carbon is so short that the growth of θ-Fe_3_C particles is restricted before the α′-to-γ transition.

We have succeeded at capturing the nucleation of austenite and following its transformation kinetics during rapid heating. The newly made γ nuclei have a lower dislocation density than α′. However, the dislocation density reaches its maximum value around Ac_3_. It is assumed that the α′/γ interface enhances screw dislocations because of the accommodation of lattice misfits, whereas the migration of the α′/γ interface is inhibited by the effect of ultrafast heating.

## Conclusions

A new *in*-*situ* XFEL measurement technique integrated with XRD line-profile analysis has been developed and applied to the steel-science field for the first time. We performed operand XRD measurements of Fe-0.1mass%C-2mass%Mn undergoing transformation from cold-rolled α′ to γ at ultrahigh heating rates above 10^4^ °C s^−1^. Dynamic changes in carbon concentrations and dislocation densities were successfully evaluated on a timescale shorter than 0.1 s during rapid heating.

Rapid heating creates ultra-fine austenite grains in cold worked martensitic steel, which is due to the enhanced nucleation of austenite in the ferrite matrix, where screw dislocation density is kept high and the super-saturated carbon concentration is maintained until the transformation from ferrite to austenite is complete during rapid heating. We note that the screw dislocation density and carbon concentration in the newly created austenite are comparable to those in the prior ferrite, which suggests that, under rapid heating, the ferrite-to-austenite transformation is due to the rearrangement of atoms in the short range. In other words, the present study captured for the first time the massive-like reverse transformation in carbon steels.
